# Shared Mechanisms in *Pparγ1sv* and *Pparγ2* Expression in 3T3-L1 Cells: Studies on Epigenetic and Positive Feedback Regulation of *Pparγ* during Adipogenesis

**DOI:** 10.1155/2024/5518933

**Published:** 2024-06-07

**Authors:** Yasuhiro Takenaka, Yoshihiko Kakinuma, Masaaki Ikeda, Ikuo Inoue

**Affiliations:** ^1^ Department of Bioregulatory Science Graduate School of Medicine Nippon Medical School, Tokyo, Japan; ^2^ Department of Diabetes and Endocrinology Saitama Medical University, Saitama, Japan; ^3^ Department of Physiology Saitama Medical University, Saitama, Japan

## Abstract

We have previously reported the identification of a novel splicing variant of the mouse peroxisome proliferator-activated receptor-*γ* (*Pparγ*), referred to as *Pparγ1sv*. This variant, encoding the PPAR*γ*1 protein, is abundantly and ubiquitously expressed, playing a crucial role in adipogenesis. *Pparγ1sv* possesses a unique promoter and 5′ untranslated region (5′UTR), distinct from those of the canonical mouse *Pparγ1* and *Pparγ2* mRNAs. We observed a significant increase in DNA methylation at two CpG sites within the proximal promoter region (-733 to -76) of *Pparγ1sv* during adipocyte differentiation. Concurrently, chromatin immunoprecipitation-quantitative PCR (ChIP-qPCR) using antibodies against H3K4me3 and H3K27ac indicated marked elevations in both methylation and acetylation of histone H3, while the repressive histone mark H3K9me2 significantly decreased, at the transcription start sites of both *Pparγ1sv* and *Pparγ2* following differentiation. Knocking down *Pparγ1sv* using specific siRNA also led to a decrease in *Pparγ2* mRNA and PPAR*γ*2 protein levels; conversely, knocking down *Pparγ2* resulted in reduced *Pparγ1sv* mRNA and PPAR*γ*1 protein levels, suggesting synergistic transcriptional regulation of *Pparγ1sv* and *Pparγ2* during adipogenesis. Furthermore, our experiments utilizing the CRISPR-Cas9 system identified crucial PPAR*γ*-binding sites within the *Pparγ* gene locus, underscoring their significance in adipogenesis. Based on these findings, we propose a model of positive feedback regulation for *Pparγ1sv* and *Pparγ2* expression during the adipocyte differentiation process in 3T3-L1 cells.

## 1. Introduction

The peroxisome proliferator-activated receptors (PPARs) are key nuclear receptors that regulate the expression of genes critical for metabolic homeostasis. Among them, PPAR*γ*, the third member of the PPAR gene subtype, is a pivotal regulator of adipogenesis [1]. PPAR*γ* modulates the transcription of essential adipocyte differentiation genes, such as *Fabp4* and *Cebpa*, in a ligand-dependent manner [2]. Two isoforms of PPAR*γ* are known: the ubiquitously expressed PPAR*γ*1 and the adipocyte-specific PPAR*γ*2, with the latter being 30 amino acids longer at the N-terminus in mice [3, 4]. In addition to these, we have identified novel human [5] and mouse *Pparγ* splicing variants [6]. *Pparγ1sv*, a mouse *Pparγ* splicing variant, is indispensable for adipocyte differentiation of 3T3-L1 and mouse primary cultured preadipocytes [6]. Notably, *Pparγ1sv* is significantly upregulated during adipocyte differentiation in these cells, and its knockdown via specific siRNAs substantially inhibits adipogenesis in 3T3-L1 cells. *Pparγ1sv* is ubiquitously expressed, with high levels in many tissues including the placenta and embryo [6, 7]. *Pparγ1sv*-knockout (KO) mice are viable and fertile but exhibit a prolonged lower body weight during and after the weaning period [7].

*Pparγ1sv* possesses unique promoter and 5′UTR sequences, providing distinct regulatory elements and a specific transcriptional initiation site for its expression. However, we found that the promoters of both *Pparγ1sv* and *Pparγ2* are similarly transactivated by C/EBP*β* and C/EBP*δ* in 3T3-L1 cells [6]. Furthermore, specific knockdown of C/EBP*β* resulted in the concomitant reduction of both mRNA and protein levels for *Pparγ1sv* and *Pparγ2*. These results imply that the expression of *Pparγ1sv* and *Pparγ2* is synergistically regulated during adipogenesis.

In 3T3-L1 cells, the *Pparγ2* promoter undergoes progressive demethylation of methylated CpG sites upon the induction of differentiation [8]. In human colorectal cancers, specific DNA methylation in a region of the *Pparγ1* promoter correlates with reduced PPAR*γ* expression [9]. In addition to DNA methylation, histone modifications also play a crucial role in the transcriptional regulation of the *Pparγ* gene [10]. In this study, we demonstrate that the epigenetic regulation of *Pparγ1sv* during adipogenesis in 3T3-L1 cells predominantly involves histone modifications, with limited DNA methylation. Furthermore, we have identified PPAR*γ*-binding sites within the *Pparγ* gene locus, suggesting the presence of a positive feedback mechanism regulating synergistic *Pparγ1sv* and *Pparγ2* expression during adipocyte differentiation.

## 2. Experimental Procedures

### 2.1. Cell Culture and Differentiation

3T3-L1 cells were obtained from JCRB Cell Bank (Osaka, Japan). Culture condition, media, and method of adipocyte differentiation were described elsewhere [6].

### 2.2. Bisulfite Sequencing

Genomic DNA was isolated from 3T3-L1 preadipocytes (day 0) and differentiated adipocytes (day 5) using DNeasy Tissue Kit (Qiagen). Two micrograms of each DNA sample was bisulfite modified using EpiTect Bisulfite Kit (Qiagen). The *Pparγ1sv* promoter region was amplified with EpiTaq HS polymerase (Takara Bio) using the following primers: Novelpro_UP3bis, 5′-TGTGATAGATAAGGTGATAGAGTTTGG-3′, and Novelpro_LP1bis, 5′-TCCCTTATATAAAAACAACCCAAACTA-3′. PCR fragments were cloned into the pGEM-T Easy Vector (Promega) and sequenced for both strands. Bisulfite sequences from all clones were analyzed using QUMA [11].

### 2.3. Histidine Modification Analyses

3T3-L1 cells cultured in 10 cm dishes were fixed with 1% formaldehyde at around 25°C for 10 min, after which fixation was halted by addition of glycine solution. Immunoprecipitated protein/DNA complexes were prepared using the Magna ChIP A kit (Millipore) following manufacturer's instructions. Anti-acetyl-histone H3 (Lys27) and anti-monomethyl-histone H4 (Lys20) antibodies were purchased from Millipore (#CS200551 and #CS200569, respectively). Anti-trimethyl-histone H3 (Lys4) and anti-dimethyl-histone H3 (Lys9) antibodies were purchased from Wako Pure Chemical Industries (#301-34811 and #304-32363, respectively). ChIP samples were analyzed by qPCR with gene-specific primers as follows: NVpro_ChIP_UP1, 5′-GTCGGAGGGTGGGGAGGAGGATG-3′, and NVpro_ChIP_LP1, 5′-CCCAATCCCAAGCCATAAAGCAC-3′, for *Pparγ1sv* promoter; NVpro_ChIP_UP3, 5′-GGAGCAAGGCGGCCAGGTAACCA-3′, and NVpro_ChIP_LP3, 5′-GGCGGGTGCTGTGCGTCGGTGAG-3′, for *Pparγ1* promoter; and g2pro_ChIP_UP2, 5′-GCCTTTATTCTGTCAACTATTCCTTTT-3′, and g2pro_ChIP_LP2, 5′-AGTATTTATCTTTGGTTGAAACTCCTA-3′, for *Pparγ2* promoter.

### 2.4. siRNA and qPCR

All siRNAs (Stealth RNAi) were purchased from Life Technologies (Carlsbad, CA, USA). Target sequences of *Pparγ1sv*, *Pparγ2*, and *Pparγ* common mRNAs were as follows: 5′-GAUCUGAAGGCUGCAGCGCUAAAUU-3′ (si*γ*1sv22), 5′-CCAGUGUGAAUUACAGCAAAUCUCU-3′ (si*γ*2_8), and 5′-CCAGGAGAUCUACAAGGACUUGUAU-3′ (si*γ*Common1). For siRNA transfection, 5 × 10^5^ 3T3-L1 cells per well were plated onto six-well plates and transfected using Lipofectamine RNAiMAX (Thermo Fisher Scientific, Waltham, MA, USA) according to the manufacturer's instructions. To quantify *Pparγ1sv* and *Pparγ2* mRNA levels, total RNA was isolated from preadipocyte and adipocyte-differentiated 3T3-L1 cells using ISOGEN (Nippon Gene, Tokyo, Japan), reverse-transcribed using SuperScript III (Life Technologies), and amplified using Thunderbird SYBR qPCR mix (Toyobo, Osaka, Japan) in an ABI Prism 7900HT sequence detection system (Life Technologies). Primer sequences for *Pparγ1sv*, *Pparγ2*, and *18S* ribosomal RNA (reference gene) qPCR analyses have been described elsewhere [6].

### 2.5. Immunoblot Analyses

3T3-L1 cells cultured in 6-well plates were lysed using RIPA buffer (20 mM Tris-HCl, pH 7.4, 100 mM NaCl, 0.5% Nonidet P-40, 0.05% SDS, and 1 mM EDTA, with protease inhibitors), briefly sonicated, and then centrifuged. The protein concentrations of the supernatants were determined using the Quick Start Bradford dye (Bio-Rad). Whole cell extracts (15 *μ*g) were mixed with 5 × SDS sample buffer containing 2-mercaptoethanol, heated for 3 min at 95°C, and then loaded onto a 12% SDS-polyacrylamide gel (SDS-PAGE). Following electrophoresis, proteins were transferred to PVDF membrane and blocked with 1% Western Blocking Reagent (Sigma-Aldrich) at 25°C for 1 h. The membrane was then incubated overnight at 16°C with antibodies against PPAR*γ* (A3409A, Perseus Proteomics), C/EBP*β* (sc-150, Santa Cruz Biotechnology), or GAPDH (G8795, Sigma-Aldrich). Chemiluminescence signals were visualized using ImmunoStar LD (Fujifilm Wako) and detected with an ImageQuant800 (Cytiva). The intensity of the protein bands was quantified using Fiji/ImageJ2.

### 2.6. PPAR*γ* ChIP-qPCR Analyses

The 3T3-L1 cells cultured in 10 cm dishes were fixed with 1% formaldehyde in PBS at 25°C for 10 min, after which fixation was halted by addition of glycine solution. Immunoprecipitated protein-DNA complexes were prepared using the Magna ChIP A kit (Millipore) according to the manufacturer's instructions. Genomic DNA was immunoprecipitated with anti-PPAR*γ* antibody (A3409A, Perseus Proteomics) or normal mouse IgG (Millipore, 12-371), purified, and amplified using Thunderbird SYBR qPCR Mix (Toyobo) in an ABI Prism 7900HT sequence detection system (Life Technologies) using the following site-specific primers: PPARg_ChIP1_UP2, 5′-GTACTTTTCTTTCTGGGTTTATTTTG-3′, and PPARg_Site1-5′_LP1, 5′-CTAATCTGGGTTAAGAGGATGTAATG-3′, for binding site 1; PPARg_ChIP_UP2, 5′-GGCTCAAAATACCCCTTCCATCTTA-3′, and PPARg_ChIP_LP2, 5′-GATGCTCAGGATTTGATGTCTCATA-3′, for binding site 2; PPARg_ChIP_UP3, 5′-CAGGTTTGATTCCCAACTCCCACATA-3′, and PPARg_ChIP_LP3, 5′-GATGATAGGCTACTTGTGAGCAAAGG-3′, for binding site 3; and g2pro_ChIP_UP2, 5′-GCCTTTATTCTGTCAACTATTCCTTTT-3′, and g2pro_ChIP_LP2, 5′-AGTATTTATCTTTGGTTGAAACTCCTA-3′, for non-PPAR*γ*-binding site.

### 2.7. CRISPR/Cas9 Genome Editing

The CRISPR guide RNAs were cloned into the CMV-hspCas9-H1-gRNA SmartNuclease vector (System Biosciences, Palo Alto, CA, USA). The Cas9-target sequences for PPAR*γ*-binding sites are as follows: 5′-CCTTCTAGCAGATCAAAAGT-3′ for site 1, 5′-TCAAAGAGTAAACCCACCAA-3′ for site 2, and 5′-ACACTGTGATGCAGAGATGC-3′ for site 3. The upstream and downstream regions adjacent to the binding site were amplified using Advantage 2 Polymerase (Takara Bio) from 3T3-L1 genomic DNA and cloned into the HR110-PA-1 vector (System Biosciences). Primer sequences to amplify the 5′- and 3′-side genomic fragments for homologous recombination are as follows: PPARg_Site1-5′_UP1, 5′-CCTCGCTCCTGTTTTCTTTGTAAATA-3′, and PPARg_Site1-5′_LP1, 5′-CTAATCTGGGTTAAGAGGATGTAATG-3′, for 5′-side of site 1; PPARg_Site1-3′_UP1, 5′-CATGAAGAAGAAAGCAACCTCTGTATAA-3′, and PPARg_Site1-3′_LP1, 5′-CCTCCCCTCCCTTCTAATAAATGCTC-3′, for 3′-side of site 1; PPARg_Site2-5′_UP1, 5′-AGCTGGACTTTTGAGTTTTTCTCTAT-3′, and PPARg_Site2-5′_LP1, 5′-TTGAGCCACAAGACCAATATAAGATACA-3′, for 5′-side of site 2; PPARg_Site2-3′_UP1, 5′-TGCTAGGTAAACTGTTCGCCACTGAG-3′, and PPARg_Site2-3′_LP1, 5′-TGGTCGGGGTAAATCCTGTCTATG-3′, for 3′-side of site 2; PPARg_Site3-5′_UP1, 5′-CAATATGCTAAGTGCTGAGTGTAATGA-3′, and PPARg_Site3-5′_LP1, 5′-GTGCTCTTAACTGCTAAGCCATCTCTC-3′, for 5′-side of site 3; and PPARg_Site3-3′_UP1, 5′-CCCACCATCATCTTGAGTGTCACATA-3′, and PPARg_Site3-3′_LP1, 5′-TTCCCCCAACCCTAAGCATTTCAACA-3′, for 3′-side of site 3. The Cas9 and targeting vectors were cotransfected into 3T3-L1 cells using PEI MAX™ (Cosmo Bio, Tokyo, Japan). For negative control clones, empty targeting vector was transfected into 3T3-L1 cells. After two days, the culture medium was replaced with DMEM containing 2.5 *μ*g/mL puromycin, and the cells were cultured for two weeks. Single colonies were isolated using cell cloning cylinders and expanded for an additional week. Heterozygous KO clones of the target site were screened by PCR.

### 2.8. Intracellular TG Content

Adipocyte differentiation was evaluated by staining cellular lipid droplets with the AdipoRed Assay Reagent (Lonza, Basel, Switzerland). The fluorescence intensity (excitation/emission485/535 nm) was measured using a Varioskan Flash (Thermo Fisher Scientific).

### 2.9. Statistical Analyses

We conducted one-way ANOVA followed by Tukey's multiple comparison test or the two-tailed unpaired *t*-test. Differences between groups were considered significant at *p* < 0.05. All data were analyzed using GraphPad Prism 5.0 (GraphPad Software, San Diego, CA, USA) and are presented as the mean (SD) of the obtained values.

## 3. Results

### 3.1. Methylation at Two CpG Sites in the Upstream Region of the *Pparγ1sv* Transcription Initiation Site Is Enhanced during Adipocyte Differentiation

**Figure 1:**
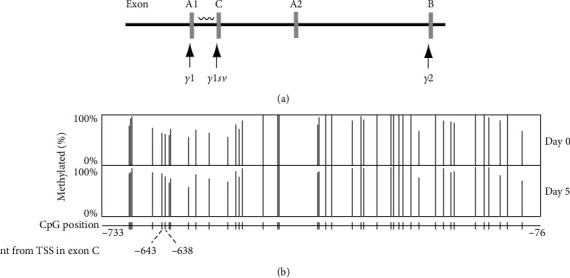
DNA methylation of *Pparγ1sv* promoter during adipogenesis. (a) The wave line represents the region analyzed by bisulfite sequencing. Arrows indicate the transcription start sites of three *Pparγ* transcripts. (b) Percentages of DNA methylation of 42 CpG islands within the 658 bp region of the *Pparγ1sv* promoter were determined through bisulfite sequencing of 90 clones at day 0 and 88 clones at day 5. Two dot lines indicate CpG sites that exhibited significant DNA methylation during adipocyte differentiation (day 5) compared to undifferentiated cells (*p* < 0.05).

*Pparγ1sv* exhibited a remarkable upregulation as early as day 3 following the induction of adipogenesis in 3T3-L1 cells [6]. To explore the regulatory mechanisms of *Pparγ1sv* transcription, we investigated the DNA methylation of CpG sites within the promoter region of *Pparγ1sv*. The methylation status of 42 CpG sites located within the upstream region spanning -733 to -76 nucleotides (nt) relative to the transcription start site (TSS) located in exon C (Figure 1(a), indicated by a wave line) was analyzed using bisulfite genomic sequencing. A comparison of the methylation percentages before (day 0) and after (day 5) differentiation in 3T3-L1 cells showed that two CpG sites, at positions -643 and -638 from TSS (Figure 1(b), indicated by dot lines), were significantly methylated in differentiated cells compared to undifferentiated cells (*p* < 0.05). However, the remaining 40 CpG sites did no undergo notable methylation or demethylation during the process of adipocyte differentiation.

### 3.2. Histone H3 Methylation and Acetylation at the Upstream Region of *Pparγ1sv* Transcription Start Site Are Elevated during Adipocyte Differentiation

**Figure 2:**
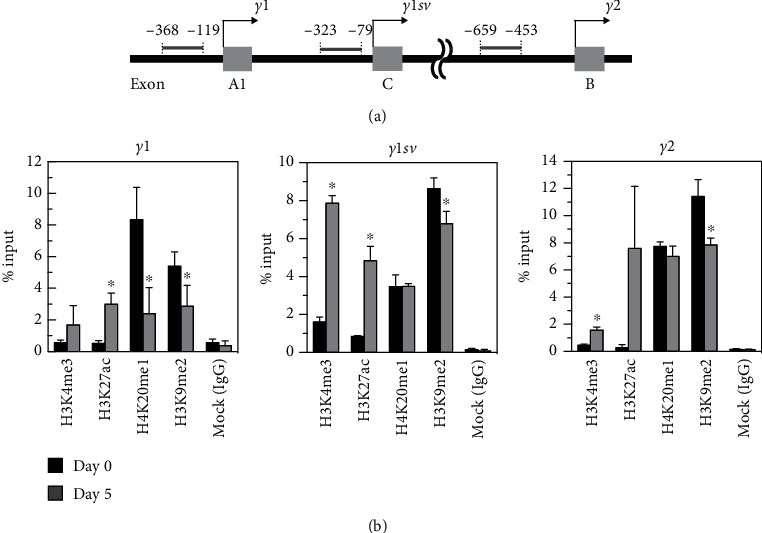
Histone modifications at the *Pparγ1sv* promoter. The promoter regions of *Pparγ1*, *Pparγ1sv*, and *Pparγ2* were analyzed for H3K4me3, H3K27ac, H4K20me1, and H3K9me2 modifications using ChIP-qPCR with specific primers. (a) Arrows indicate positions of transcription start sites of the three transcription variants. The gray bars mark the analyzed promoter regions, with distances from each transcription start site indicated in nucleotides. (b) Enrichment of the *Pparγ1sv* promoter region by ChIP using antibodies specific to H3K4me3, H3K27ac, H4K20me1, or H3K9me2 on days 0 and 5 of adipocyte differentiation. The values represent means (SD) from three separate experiments. An asterisk (∗) indicates *p* < 0.05 by the two-tailed unpaired *t*-test for day 0 vs. day 5.

Increases in histone modification levels at the *Pparγ* promoter have been associated with adipocyte differentiation [12, 13]. We examined the trimethylation of histone H3 lysine 4 (H3K4me3), acetylation of histone H3 lysine 27 (H3K27ac), monomethylation of histone H4 lysine 20 (H4K20me1), and dimethylation of histone H3 lysine 9 (H3K9me2) at the *Pparγ* promoters (Figure 2(a)). Previous report indicated that H3K4me3 and H3K27ac levels were elevated in the region spanning the TSSs [14]. For H4K20me1, an increase was observed in the downstream regions of the transcription start sites for both *Pparγ1* and *Pparγ2* [12]. In our study, ChIP-qPCR with specific primers revealed remarkable increases in H3K4me3 and H3K27ac modifications at the upstream regions of the TSSs for *Pparγ1sv* and *Pparγ2* on day 5 of adipocyte differentiation (Figure 2(b)), indicating active transcription of these genes. This was further supported by substantial demethylation of H3K9me2, a repressive histone marks, at the promoter regions of *Pparγ1sv* and *Pparγ2*. Meanwhile, the levels of H4K20me1, associated with DNA damage repair or DNA replication, remained largely unchanged.

### 3.3. Synergistic Regulation of Gene Expression between *Pparγ1sv* and *Pparγ2*

**Figure 3:**
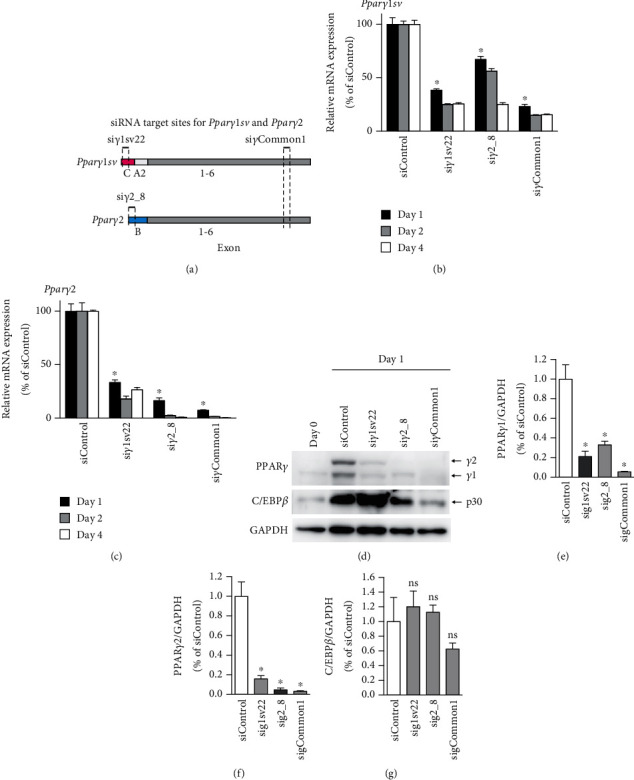
Synergistic expression of *Pparγ1sv* and *Pparγ2* during adipocyte differentiation. (a) Target sites for siRNAs against *Pparγ1sv* (si*γ*1sv22), *Pparγ2* (si*γ*2_8), and both transcripts (si*γ*Common1). (b, c) Relative mRNA levels of (b) *Pparγ1sv* and (c) *Pparγ2* in siRNA-treated 3T3-L1 cells at days 1, 2, and 4 of adipocyte differentiation were determined by qPCR. Expression levels were normalized to 18S rRNA. Values are shown as mean (SD) (*n* = 3). An asterisk (∗) denotes a significant difference (*p* < 0.05) compared to the siControl group for day 1 samples only. (d) Immunoblot analyses of PPAR*γ* (*γ*1 and *γ*2), C/EBP*β* (p30), and GAPDH, for undifferentiated (day 0) and siRNA-treated 3T3-L1 cells at day 1. (e–g) Quantification of immunoblot band intensities for (e) PPAR*γ*1, (f) PPAR*γ*2, and (g) C/EBP*β*, normalized to GAPDH levels. Relative values are presented as mean (SD) (*n* = 3). An asterisk (∗) denotes a significant difference (*p* < 0.05) compared to the siControl group, as determined by one-way ANOVA with Tukey's multiple comparison test. ns: not significant.

We next investigated whether the specific knockdown of either *Pparγ1sv* or *Pparγ2* would influence the expression of the other. Using a siRNA (si*γ*1sv22) we designed (Figure 3(a)), which specifically targets *Pparγ1sv* transcripts in 3T3-L1 cells [6] (Figure 3(b)), we observed a concurrent decrease in *Pparγ2* mRNA levels (Figure 3(c)) during adipocyte differentiation, as measured by quantitative PCR (qPCR). Similarly, a siRNA specific to *Pparγ2* (si*γ*2_8) led to a significant reduction in *Pparγ1sv* levels (Figure 3(b)). We then assessed whether the protein levels of PPAR*γ*1 and PPAR*γ*2, which are derived from *Pparγ1sv* and *Pparγ2*, respectively, were influenced by treatment with their specific siRNAs. Indeed, knocking down *Pparγ1sv* with si*γ*1sv22 significantly reduced the levels of both PPAR*γ*1 and PPAR*γ*2 proteins on day 1 (Figures 3(d), 3(e), and 3(f)); similarly, silencing *Pparγ2* with si*γ*2_8 led to decreased levels of both PPAR*γ*1 and PPAR*γ*2 proteins (Figures 3(d), 3(e), and 3(f)), whereas C/EBP*β* (p30), the major isoform of C/EBP*β* [6] that is transiently expressed at the early stages of adipocyte differentiation [3], was similarly upregulated in cells transduced with siControl, si*γ*1sv22, and si*γ*2_8 (Figures 3(d) and 3(g)), indicating that the induction of adipogenesis was effectively achieved. These findings imply the existence of synergistic regulatory mechanisms for the gene expression of *Pparγ1sv* and *Pparγ2* and their protein products, PPAR*γ*1 and PPAR*γ*2, during adipocyte differentiation.

### 3.4. PPAR*γ*-Binding Sites within the *Pparγ* Gene Locus Regulate *Pparγ1sv* and *Pparγ2* Expression and Adipogenesis

**Figure 4:**
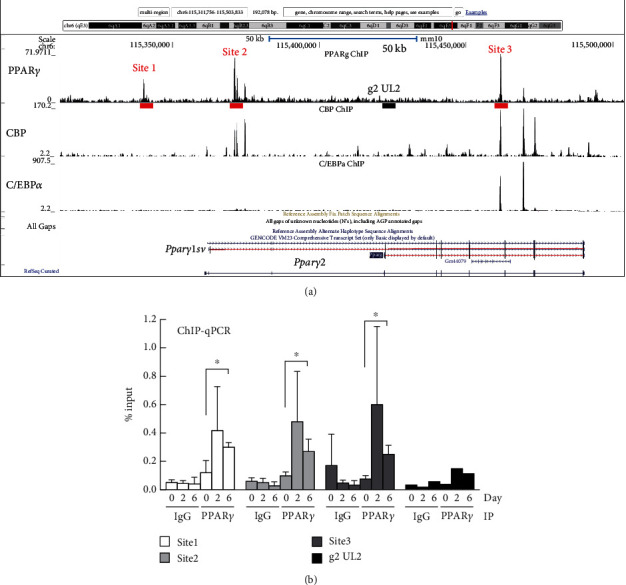
Potential binding sites of PPAR*γ* protein in the *Pparγ* gene locus. (a) Identified binding sites of PPAR*γ*, CREB-binding protein (CBP), and C/EBP*α* in the *Pparγ* gene locus, as indicated by the densities from ChIP-seq data (NCBI GSE20752). Sites 1–3 are candidate PPAR*γ*-binding sites, while g2 UL2 is a non-PPAR*γ*-binding site. (b) ChIP-qPCR assay with anti-PPAR*γ* antibody and normal mouse IgG was performed to evaluate the binding of PPAR*γ* protein to candidate sites 1–3 at days 0, 2, and 6 of 3T3-L1 adipocyte differentiation. Values are expressed as mean (SD) (*n* = 4). An asterisk (∗) indicates *p* < 0.05 by the two-tailed unpaired *t*-test for day 0 vs. day 6.

**Figure 5:**
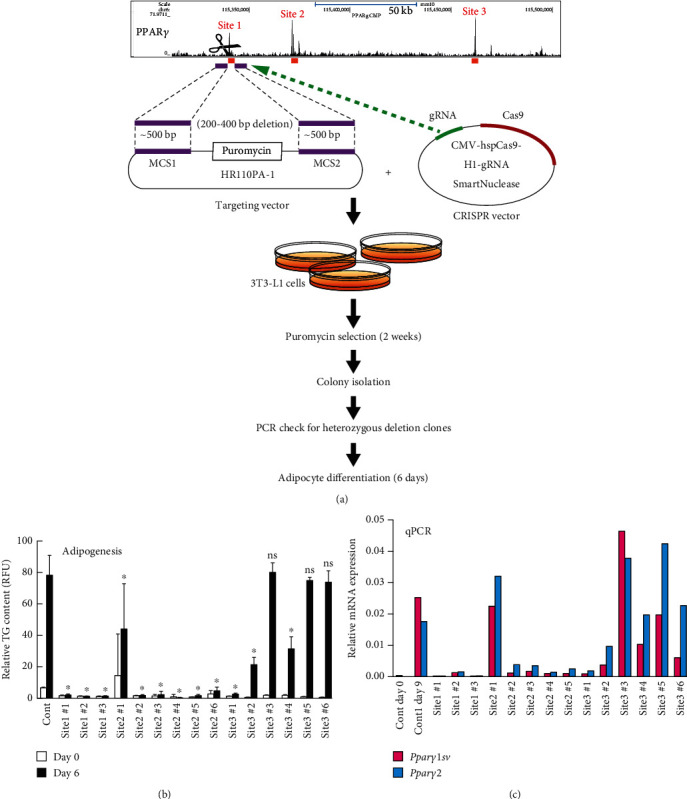
PPAR*γ*-binding sites 1 and 2 are crucial to the expression of *Pparγ1sv*, *Pparγ2*, and adipogenesis in 3T3-L1 cells. (a) Knockout (KO) strategy for PPAR*γ*-binding sites 1–3 using the CRISPR-Cas9 with targeting vectors. (b) Relative triglyceride content (TG) in 3T3-L1 control cells and sites 1–3 KO clones was evaluated on days 0 and 6 of adipocyte differentiation. Values are shown as mean (SD) (*n* = 4). An asterisk (∗) indicates a significant difference (*p* < 0.05) compared to control cells on day 6. ns: not significant. (c) Relative *Pparγ1sv* and *Pparγ2* mRNA levels in control and sites 1–3 KO clones on day 6 of adipocyte differentiation. Values were obtained by averaging the results from two independent experiments.

To elucidate the synergistic gene regulatory mechanisms between *Pparγ1sv* and *Pparγ2*, we explored potential cis-acting elements within or adjacent to the *Pparγ* gene locus. Initially, we analyzed NCBI GEO datasets for a chromatin immunoprecipitation (ChIP) sequencing study with an anti-PPAR*γ* antibody [15]. Based on this analysis, we found three potential PPAR*γ*-binding sites (sites 1–3) (Figure 4(a)). The association of CREB-binding protein (CBP) with sites 2 and 3, and C/EBP*α* with site 3, supports the notion that these are possible binding sites of transcription regulators. We validated the notable binding of PPAR*γ* protein to all three sites during adipocyte differentiation using a ChIP-qPCR assay with an anti-PPAR*γ* antibody (Figure 4(b)). To further demonstrate the significance of these sites in adipocyte differentiation and the regulation of *Pparγ* expression, we established heterozygous KO-3T3-L1 cells lacking each of three binding sites using CRISPR-Cas9 system with the targeting vectors (Figure 5(a)). During adipocyte differentiation, the accumulation of intracellular triglyceride (TG) was significantly reduced in the sites 1 and 2 KO clones, while the effect was less pronounced in the site 3 KO clones (Figure 5(b)), suggesting that sites 1 and 2 are critical for adipogenesis. Consistent with TG accumulation patterns, *Pparγ1sv* and *Pparγ2* mRNA levels were largely suppressed in the sites 1 and 2 KO clones on day 9 of differentiation, whereas levels in the site 3 KO clones were comparable with those in control cells (Figure 5(c)).

## 4. Discussion

Previous studies have demonstrated that in murine 3T3-L1 cells, methylated CpG sites on the *Pparγ2* promoter undergo progressive demethylation during adipogenesis [8]. Similarly, study on human colorectal cancer has revealed an inverse correlation between methylation of specific regions in the *Pparγ1* promoter and PPAR*γ* expression [9]. However, in our studies, DNA methylation levels of the *Pparγ1sv* promoter remained largely unchanged during adipogenesis in 3T3-L1 cells (Figure 1(b)). This suggests that DNA methylation may not play a significant role in the epigenetic regulation of *Pparγ1sv* expression. Instead, our findings indicate that *Pparγ1sv* and *Pparγ2* transcripts are likely regulated through a common mechanism involving histone modifications. ChIP-qPCR with antibodies against H3K4me3 and H3K27ac showed significant elevations in these histone modifications at the regions close to transcription start sites of both *Pparγ1sv* and *Pparγ2* upon differentiation (Figure 2(b)). Although H3K4me3 and H3K27ac levels also increased at the *Pparγ1* promoter, the extent was comparatively smaller, which may be indicative of a limited enhancement of *Pparγ1* mRNA expression during adipogenesis in 3T3-L1 cells.

Positive feedback mechanisms play vital regulatory roles in the expression of genes required for proper cellular differentiation, proliferation, and metabolic homeostasis [16]. Previous studies have suggested the involvement of PPAR*γ*-binding sites within the *Pparγ* gene locus [13, 15] and feedback regulation of *Pparγ* expression by PPAR*γ* protein in adipogenesis [10, 12]. These studies primarily focused on *Pparγ2* expression, a key regulator of adipogenesis. In this study, we revealed that the specific knockdown of either *Pparγ1sv* or *Pparγ2* leads to the reciprocal downregulation of each transcript (Figures 3(b) and 3(c)) and their protein products (Figures 3(d), 3(e), and 3(f)). Furthermore, we demonstrated that two out of three identified PPAR*γ*-binding sites within the *Pparγ* gene locus are critical for both adipocyte differentiation (Figure 5(b)) and *Pparγ* expression (Figure 5(c)) in 3T3-L1 cells. In contrast, while PPAR*γ* binding was observed at site 3, this site does not appear to be significantly involved in *Pparγ* expression or the subsequent adipocyte differentiation. This could be due to site 3 being located considerably downstream of the TSSs (Figure 4(a)). Therefore, the binding of PPAR*γ* to site 3 might be involved in the regulation of other gene expressions by PPAR*γ*, but this aspect will be clarified in future studies.

In conclusion, we have shown that the promoter regions of two splicing variants, *Pparγ1sv* and *Pparγ2*, undergo similar histone modifications during adipogenesis. Furthermore, the expression of these variants mutually influences each other's levels, potentially regulated by the binding of their protein products, PPAR*γ*1 and PPAR*γ*2, to shared regions in the *Pparγ* gene. Given that these sites are consistent between 3T3-L1 cells and mouse primary adipocytes [17], we propose that our findings can be applied to *in vivo* studies to gain a more comprehensive understanding of *Pparγ* expression regulation during adipogenesis.

## Data Availability

The data that support the findings of this study are available from the corresponding author upon reasonable request.
